# Prenatal Benzydamine Exposure Induces Fetal Growth Restriction and Maternal Oxidative Stress in Rats

**DOI:** 10.3390/ijms27073005

**Published:** 2026-03-26

**Authors:** Bianca-Eugenia Ősz, Ruxandra Ștefănescu, Amelia Tero-Vescan, Camil-Eugen Vari, George Jîtcă, Erzsébet Májai, Andreea Sălcudean

**Affiliations:** 1 Department of Pharmacology and Clinical Pharmacy, Faculty of Pharmacy, George Emil Palade University of Medicine, Pharmacy, Science, and Technology of Targu Mures, 540142 Targu Mures, Romania; bianca.osz@umfst.ro (B.-E.Ő.); camil.vari@umfst.ro (C.-E.V.); george.jitca@umfst.ro (G.J.); 2 Department of Pharmacognosy and Phytotherapy, Faculty of Pharmacy, George Emil Palade University of Medicine, Pharmacy, Science, and Technology of Targu Mures, 540142 Targu Mures, Romania; 3 Department of Biochemistry, Faculty of Medicine in English, George Emil Palade University of Medicine, Pharmacy, Science, and Technology of Targu Mures, 540142 Targu Mures, Romania; amelia.tero-vescan@umfst.ro; 4 Department of Toxicology and Biopharmacy, Faculty of Pharmacy, George Emil Palade University of Medicine, Pharmacy, Science, and Technology of Targu Mures, 540142 Targu Mures, Romania; erzsebet.fogarasi@umfst.ro; 5 Department of Bioethics, Deontology and Medical Communication, Faculty of Medicine, George Emil Palade University of Medicine, Pharmacy, Science, and Technology of Targu Mures, 540142 Targu Mures, Romania; andreea.salcudean@umfst.ro

**Keywords:** benzydamine, prenatal exposure, maternal toxicity, oxidative stress, fetal growth restriction

## Abstract

Benzydamine is a nonsteroidal anti-inflammatory drug widely used in topical formulations but occasionally misused orally at high doses for psychoactive effects. Data regarding the safety of benzydamine at supratherapeutic doses are limited and mainly focus on central nervous system effects. Even less information is available concerning its safety during pregnancy, despite the increased risk of unplanned pregnancies among users of psychoactive substances. In this preliminary study, we aimed to evaluate the maternal and fetotoxic potential of benzydamine to support future targeted reproductive toxicity investigations. Pregnant Wistar rats received benzydamine throughout gestation, followed by cesarean section and evaluation of fetal viability, fetal body weight at term, and macroscopic abnormalities. Maternal biochemical parameters related to hepatic, renal, and metabolic function, and oxidative stress markers, were also assessed. Results were compared with those of a control group. No significant differences in routine biochemical parameters were observed between groups; however, benzydamine exposure was associated with reduced fetal body weight and increased maternal plasma malondialdehyde levels. These findings suggest that benzydamine may impair fetal growth through indirect maternal toxicity and oxidative stress rather than direct teratogenic effects.

## 1. Introduction

Benzydamine, marketed over the counter (OTC) primarily as its hydrochloride salt, is an indole-derived nonsteroidal anti-inflammatory drug (NSAID) that also exhibits local anesthetic, analgesic, antimicrobial, and antifungal properties [1]. It is formulated for topical administration in the treatment of oropharyngeal and gynecological disorders and is approved for pediatric use in oropharyngeal conditions. Despite its intended topical application, there are reports of oral misuse at supratherapeutic systemic doses for recreational purposes, which has been associated with the onset of hallucinations and nonspecific sensory disturbances [2,3,4,5,6].

The pharmacokinetic profile of benzydamine varies with the route of administration. Following oral dosing, the drug is rapidly absorbed, achieving peak plasma concentrations within a short time and exhibiting an elimination half-life of approximately 8–13 h, with low plasma protein binding (<20%). In contrast, systemic absorption after topical, dermal, or mucosal administration is limited, resulting in high local drug concentrations but minimal systemic exposure. Benzydamine is extensively metabolized via oxidation, conjugation, and dealkylation. Overall, its low systemic absorption through skin and non-specialized mucosae contributes to a reduced risk of systemic adverse effects when administered by these routes [7,8]. Gastrointestinal symptoms such as nausea, vomiting, and epigastric pain are common and resemble NSAID-related toxicity. No benzydamine-associated fatalities have been reported, and hypersensitivity remains the sole documented contraindication [2].

The use of benzydamine, even on an occasional basis, may increase the risk of unprotected sexual activity and unintended pregnancy, like other psychoactive substances [9,10].

Despite its widespread availability as an OTC-drug, data regarding the safety of benzydamine use during pregnancy are lacking. In particular, the absence of reproductive toxicity studies evaluating both therapeutic topical use and high-dose oral exposure limits the ability to assess potential risks associated with benzydamine administration during gestation [11]. Isolated clinical reports have described premature constriction of the fetal ductus arteriosus following maternal benzydamine exposure [12,13], an effect consistent with the class-related action of NSAIDs during late gestation. This phenomenon is attributed to reduced prostaglandin synthesis resulting from cyclooxygenase (COX-1) inhibition [14].

Many drug-induced fetal toxicities are mediated through oxidative stress, which disrupts placental function, impairs organogenesis, and contributes to growth restriction or developmental abnormalities [15,16]. Pregnancy is physiologically characterized by increased oxidative vulnerability, particularly in the placenta and developing fetal tissues [17,18]. Therefore, biomarkers of oxidative stress are relevant indicators of potential fetal harm [19]. As pregnancy advances, remodeling of maternal spiral arteries by extravillous trophoblasts transforms them into high-capacity, low-resistance vessels, increasing placental oxygenation and blood flow, which, while enhancing oxygen delivery, also promotes reactive oxygen species generation through mitochondrial oxidative phosphorylation and enzymatic pathways [20,21,22]. Malondialdehyde (MDA) is a well-established marker of lipid peroxidation, reflecting oxidative damage to cellular membranes with possible side effects on pregnancy evolution and safety [23], while xanthine oxidase (XO) is a major enzymatic source of reactive oxygen species (ROS), generating superoxide and hydrogen peroxide during purine metabolism, with high activity indicating enhanced oxidative stress [24].

Considering the aforementioned statements, this study was designed to explore the potential maternal and fetal effects of benzydamine, with the objective of generating preliminary data to guide future targeted reproductive toxicity investigations. Furthermore, assessment of MDA levels and XO activity was performed to provide exploratory mechanistic insight into possible modulation of oxidative stress during pregnancy.

## 2. Results

All determinations were performed individually for each animal in both groups, including 13 animals in the control group and 20 animals in the benzydamine group.

### 2.1. Fetal Development

Mean fetal weight was analyzed using litter-based averages, with 20 litters in the benzydamine group and 13 litters in the control group.

The benzydamine-treated group showed a lower overall mean fetal weight compared with the control group. The distribution of fetal weights in the benzydamine group also showed greater variability, with several litters presenting markedly reduced mean weights (see Table 1).

All statistical comparisons between groups demonstrated a significant reduction in fetal weight in the benzydamine group compared with controls (*p* < 0.05), indicating that prenatal exposure to benzydamine was associated with impaired fetal growth under the experimental conditions.

Other observations

Fetal viability was also adversely affected by benzydamine treatment, as evidenced by an increased total number of resorptions (early and late) and a higher number of dead fetuses in the benzydamine-treated group compared with the control group.

**Figure 1:**
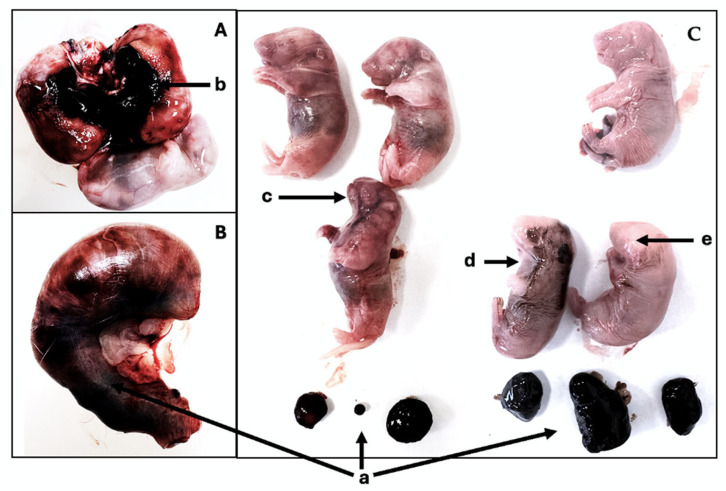
Abnormal embryos and fetuses in the benzydamine-treated group. (**A**)—one fetus located outside the yolk sac; two fetuses within the yolk sac; hemorrhage (b). (**B**)—fetus within the yolk sac; multiple resorptions (a). (**C**)—fetuses displaying various developmental abnormalities (mouth and nose—c, internal hemorrhage, including ocular hemorrhage—d, head—e).

In the control group, only one female rat presented three early resorptions and one underdeveloped fetus (Figure 1A). In contrast, the benzydamine-treated group exhibited a range of pathological findings, including intrauterine and placental hemorrhages, fetal intra-abdominal hemorrhages, and implantation restricted to a single uterine horn (Figure 1B,C).

### 2.2. Maternal Biochemical Parameters

No statistically significant differences were observed between the benzydamine-treated group and the control group for the analyzed biochemical parameters.

Nevertheless, individual animals in the benzydamine group exhibited marked deviations from control values, including elevated transaminases and glucose levels. These biochemical alterations coincided with pronounced pathological findings, such as increased fetal resorptions, intrauterine hemorrhages, placental blood clots, and fetal intraabdominal hemorrhages, which were not observed in the control group. The presence of fetal abnormalities in dams with altered biochemical parameters supports a potential relationship between maternal metabolic disturbance and adverse developmental outcomes.

The overall trend of changes in the analyzed biochemical parameters between the two groups is summarized in Table 2.

Compared with the control group, benzydamine treatment resulted in alterations in multiple biochemical parameters, indicating systemic effects.

Protein metabolism—benzydamine-treated animals exhibited lower albumin and total protein levels compared with controls, accompanied by increased globulin concentrations and a consequent reduction in the albumin/globulin ratio, suggesting altered protein metabolism and/or an inflammatory response.

Liver function markers—markers of hepatic function were decreased in the benzydamine group. Activities of AST, ALT, and ALP were lower than in controls, indicating protective effects.

Renal function markers—benzydamine administration was associated with elevated blood urea nitrogen (BUN) and an increased BUN/ALB ratio compared with controls, while creatinine levels remained relatively stable or slightly reduced. These findings suggest functional renal alterations.

In one female rat that carried a single fetus with an increased body weight (7.64 g), this outcome may be associated with elevated maternal glucose levels. In this case, glucose concentration reached 159.55 mg/dL, exceeding the mean glucose level observed in the control group (137.54 mg/dL), suggesting that maternal metabolic disturbances may have contributed to altered fetal growth.

### 2.3. Serum Malondialdehyde (MDA) Levels and Xanthine Oxidase (XO) Activity

**Figure 2:**
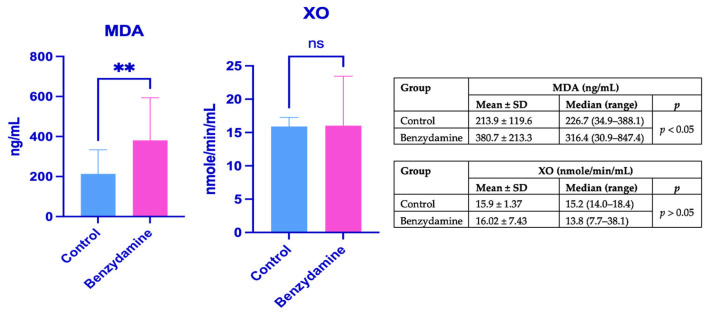
Maternal plasma malondialdehyde levels and xanthine oxidase activity (** = statistically significant; ns = non-significant).

Benzydamine administration was associated with a significant increase in serum MDA levels in pregnant rats exposed to benzydamine, whereas XO activity did not differ significantly from control values. Maternal plasma MDA concentrations and XO activity are shown in Figure 2 and are expressed as mean ± standard deviation and median (range).

When maternal plasma MDA levels were considered, visible fetal toxicity tended to be more apparent in fetuses from females with higher maternal MDA levels, although this tendency was not observed with respect to fetal weight.

## 3. Discussion

Fetal development was adversely affected by benzydamine exposure, as evidenced by reduced fetal body weight compared with controls, indicating impaired growth. In experimental rat models, low fetal weight, classified as small for gestational age (SGA) and defined as a birth weight below the 10th percentile, is a recognized indicator of fetal toxicity and reflects disrupted intrauterine development. Such growth restriction is commonly associated with maternal or placental factors and has been linked to long-term metabolic and developmental consequences in the offspring [25].

The placenta plays a critical protective role during gestation, and prenatal stress has been shown to impair placental function. Experimental studies indicate that maternal stress disrupts placental circulation, reduces placental efficiency and fetal oxygenation, and induces a hypercoagulable state. These effects are associated with downregulation of the placental PI3K/AKT/mTOR signaling pathway, leading to impaired cellular processes essential for placental barrier integrity and function, thereby contributing to abnormal fetal development [26]. Previous studies have shown that oral benzydamine administration to pregnant rats at 200 mg/kg/day during gestational days 6–15 induced marked maternal toxicity, with mortality rates of approximately 35%, and was associated with significant reductions in litter size, number of live births, and offspring body weight at birth. In contrast, exposure to doses up to 200 mg/kg during the critical period of organogenesis did not elicit maternal toxicity or adverse reproductive or teratogenic effects. These findings indicate that impaired reproductive outcomes following benzydamine exposure are largely secondary to maternal toxicity during late gestation rather than to direct teratogenic effects [12]. These findings are consistent with our results (although in our study the dosing regimen differed, being administered from GD2 throughout gestation), since no treatment-related external macroscopic malformations were observed in fetuses from the benzydamine-treated group. However, the presence of subtle structural or functional alterations cannot be excluded.

The occurrence of SGA offspring may also be attributed to placental dysfunction, which can compromise pregnancy maintenance and fetal growth. The placenta plays a central role in fetal development by regulating maternal-fetal exchange, endocrine signaling, and protection against xenobiotics. Consequently, alterations in placental structure or function represent critical indicators in the assessment of embryo-fetal toxicity [27].

Although no statistically significant differences were detected in maternal biochemical parameters, the benzydamine-treated group showed pathological alterations at the uteroplacental and fetal levels. Such findings suggest that benzydamine may induce localized or transient toxic effects that are not adequately reflected by systemic biochemical markers.

In reproductive toxicity studies, fetal resorptions and hemorrhagic lesions are considered sensitive indicators of adverse developmental effects [28], even in the absence of consistent maternal biochemical disturbances. Therefore, the pathological findings observed in the present study are biologically relevant and may indicate early or subclinical toxicity.

Although causality cannot be established, maternal hyperglycemia observed in one dam carrying a single macrosomic fetus may have influenced fetal growth by enhancing nutrient transfer to the fetus [29]. These observations support the hypothesis that benzydamine-induced metabolic disturbances in the dam may indirectly affect fetal development.

Benzydamine has been reported in older in vitro and experimental studies to exhibit antithrombotic effects, including inhibition of rat platelet aggregation induced by adenosine diphosphate (ADP) and collagen, as well as reduced venous thrombosis following oral administration in rat models [30]. Although these findings raise the possibility that altered hemostasis may have contributed to the intrauterine and placental hemorrhagic changes observed in the present study, this interpretation remains speculative. Importantly, the current study was not designed to evaluate coagulation or platelet function, and no hemostatic parameters were assessed. Therefore, targeted experimental studies are warranted to clarify whether benzydamine influences maternal or placental hemostasis and to determine the relevance of these effects during pregnancy.

Oral administration of benzydamine was associated with slightly lower levels of liver function–related biochemical markers (AST, ALT, and ALP) compared with the control group; however, these differences did not reach statistical significance. Therefore, no definitive hepatoprotective effect can be concluded. Nevertheless, the observed trends are consistent with previous experimental studies suggesting a potential role of benzydamine in attenuating hepatocellular injury, including ethanol-induced liver damage. Supporting mechanistic evidence from in vitro studies using RAW 264.7 macrophages has shown that benzydamine hydrochloride significantly suppresses pro-inflammatory cytokines (TNF-α and IL-6) while upregulating the anti-inflammatory cytokine IL-10. This favorable modulation of the inflammatory response is accompanied by a pronounced reduction in reactive oxygen species (ROS) production, stabilization of mitochondrial membrane potential, and decreased DNA fragmentation, collectively indicating a strong cytoprotective effect against ethanol-induced oxidative stress and apoptosis [31]. Furthermore, zebrafish embryo models provide compelling evidence for the protective role of benzydamine against ethanol-induced teratogenicity. Co-exposure to benzydamine restored redox homeostasis, normalized ROS levels, and reduced lipid peroxidation. In addition, benzydamine downregulated ethanol-metabolizing enzymes implicated in ethanol toxicity (CYP2Y3 and CYP3A65) and mitigated structural abnormalities, including muscle fiber disruption and apoptosis within neural tissues [32].

Benzydamine-exposed animals exhibited reduced albumin and total protein levels, accompanied by increased globulin concentrations, indicating a shift in protein balance commonly associated with inflammatory states or hepatic and renal dysfunction. Albumin, a major liver-synthesized protein essential for maintaining oncotic pressure, was decreased, suggesting either impaired hepatic synthesis [33] or increased protein loss [34]. Conversely, elevated globulin levels are typically indicative of immune activation or inflammatory responses. The resulting decrease in the albumin-to-globulin (A/G) ratio reflects a transition from a physiological, albumin-dominant profile toward a globulin-dominant state often observed in pathological conditions. If these alterations were primarily attributable to hepatic dysfunction, they could be explained by reduced albumin synthesis [33] and increased globulin production secondary to immune activation in response to liver injury [35]. However, the observation that hepatic enzyme levels were lower in the benzydamine-treated group compared with controls argues against overt hepatocellular damage. Instead, a renal origin for the altered protein profile appears more plausible, particularly considering the concomitant increases in blood urea nitrogen (BUN) and potassium levels, which may reflect impaired renal handling or protein loss. Although creatinine levels and the BUN/creatinine ratio did not differ significantly between groups, evaluation of the BUN-to-albumin ratio (BUN/ALB) revealed an increasing trend in the benzydamine-treated animals. This parameter has been proposed as a sensitive indicator of early renal dysfunction and a predictor of acute kidney injury [36], thereby reinforcing the possibility of subtle renal impairment associated with benzydamine exposure. Notably, evidence regarding benzydamine-induced nephrotoxicity remains limited, with only a single case report in the literature describing reduced glomerular filtration rate following prolonged (four-month) topical use of a 3% benzydamine-containing formulation [37]. Although creatinine levels remained stable in the present study, the increasing trend observed in the BUN/ALB ratio may provide a more sensitive indication of early or subclinical renal impairment, suggesting that this parameter could offer a more nuanced assessment of renal alterations in this experimental model.

MDA is a highly reactive aldehyde and one of the end products of lipid peroxidation resulting from oxidative degradation of polyunsaturated fatty acids in cellular membranes. As such, MDA is widely used as a biomarker of oxidative stress and cellular membrane damage. Elevated MDA levels reflect increased reactive oxygen species (ROS) generation and impaired antioxidant defense mechanisms. During pregnancy, oxidative balance is critical for normal placental function and fetal development, and excessive oxidative stress has been implicated in embryotoxicity, teratogenesis, and pregnancy complications [17]. Therefore, assessment of MDA levels in maternal and fetal tissues provides valuable insight into the potential pro-oxidant effects of pharmacological agents administered during gestation. Changes in MDA concentrations may indicate oxidative damage, altered placental transfer, or increased vulnerability of the developing fetus to lipid peroxidation [38], making MDA a sensitive and relevant indicator for evaluating the safety of drug administration during pregnancy. In the present study, benzydamine administration resulted in a significant elevation of plasma malondialdehyde levels in pregnant rats, indicating enhanced lipid peroxidation and systemic oxidative stress. In contrast, xanthine oxidase activity remained unchanged compared with control animals. This divergence suggests that benzydamine-induced oxidative damage is unlikely to be primarily mediated by xanthine oxidase–derived reactive oxygen species. Malondialdehyde reflects the cumulative end-products of polyunsaturated fatty acid peroxidation and integrates oxidative insults arising from multiple cellular sources, whereas xanthine oxidase represents a single, tightly regulated enzymatic contributor to reactive oxygen species generation [39]. Mechanistically, benzydamine may promote oxidative stress through alternative pathways, including mitochondrial dysfunction, inflammatory cell activation, or disruption of endogenous antioxidant defenses, rather than through sustained activation of xanthine oxidase. In pregnancy, physiological adaptations such as increased lipid availability, altered redox homeostasis, and heightened metabolic demand may further sensitize plasma lipids to oxidative damage, amplifying malondialdehyde formation [40] even in the absence of measurable changes in specific pro-oxidant enzymes. The timing of sample collection should also be considered when interpreting the oxidative stress markers evaluated in this study. It is possible that XO activity may have transiently increased earlier during gestation and subsequently returned to baseline levels by gestational day 21, when the samples were collected. In contrast, MDA, a stable end-product of lipid peroxidation, may persist longer in biological systems and therefore reflect the cumulative oxidative damage that occurred during earlier stages of exposure. Consequently, the observed increase in MDA levels despite unchanged XO activity may indicate that lipid peroxidation products remain detectable after the normalization of upstream enzymatic sources of reactive oxygen species.

In this context, alterations in oxidative stress markers observed in the present study may represent a mechanistic link between benzydamine exposure, placental dysfunction, and restricted fetal growth. Although previous studies have reported that prenatal benzydamine exposure reduces litter size and birth weight [12], our study further demonstrates that oxidative stress may underlie these effects, providing novel mechanistic insight into fetal growth restriction.

Taken together, these observations suggest that while benzydamine may not exert overt teratogenic effects, its potential to disrupt fetal physiology through indirect mechanisms, particularly at high doses or during sensitive periods of pregnancy, warrants further investigation.

While these findings were obtained in an animal model, they raise important concerns regarding the potential developmental risks associated with benzydamine exposure during pregnancy. Given the similarities in fundamental mechanisms of oxidative stress and placental function across mammalian species, these results may have translational relevance for humans. Consequently, caution may be warranted when considering benzydamine use during pregnancy, and further clinical and epidemiological studies are necessary to clarify its safety profile in the human population.

### Limitations

The present work represents an exploratory study, and several limitations should be acknowledged. Fetal evaluation was limited to body weight, gross abnormalities, and fetal mortality, without detailed histopathological examination of fetal organs or placental tissues, nor molecular analyses of fetal oxidative stress pathways.

Xanthine oxidase activity was assessed exclusively in plasma, which may not accurately reflect tissue-specific enzymatic changes occurring in vascular endothelium or placenta, where xanthine oxidase activity is more pronounced. Only a single time point was evaluated, limiting the ability to detect transient or early alterations in xanthine oxidase activity following benzydamine exposure. Additional markers of oxidative stress and antioxidant defense, such as superoxide dismutase, catalase, glutathione peroxidase, or total antioxidant capacity, were not measured and could provide further insight into the mechanisms underlying the observed increase in lipid peroxidation. The complex physiological redox adaptations associated with pregnancy may modulate oxidative responses to pharmacological agents, and these factors should be considered when extrapolating the findings beyond this experimental context. Moreover, the timing of maternal plasma collection (performed after C-section) limits the ability to determine whether lipid peroxidation markers were elevated throughout gestation or only at the time of sampling.

The relatively small number of subjects included may limit the statistical power of the analysis and the generalizability of the findings. Since only a single dose (261 mg/kg body weight) was evaluated, it does not allow assessment of potential dose–response relationships. Additionally, the absence of comparable data in the existing literature limits the possibility of performing a direct comparative evaluation of our results. Sex-specific analyses were also not performed. Therefore, further studies with larger sample sizes, multiple dosing regimens including therapeutic dosage, and additional comparative data are needed to better validate and extend these findings. Sex-based analyses are also required to better characterize the effects of gestational benzydamine exposure.

## 4. Materials and Methods

### 4.1. Evaluation of the Teratogenic and Fetal Toxic Potential of Benzydamine

A total of 40 female and 40 male white Wistar rats, aged 4–6 months, obtained from “George Emil Palade” University of Medicine, Pharmacy, Science and Technology of Targu Mures, Romania, Experimental Station and Biobase, were used for mating.

Throughout the study, animals were housed under standard laboratory conditions, including controlled temperature and humidity (22 ± 3 °C; 30–70% relative humidity), a 12 h light/12 h dark cycle, and ad libitum access to species-appropriate food and water [41].

Mating was performed in accordance with the rat estrous cycle. Estrous cycle stages were monitored by periodic vaginal smear examinations. Females in estrus were paired with healthy males at a 1:1 ratio, and vaginal smears were collected the following morning to confirm the presence of sperm. The day on which mating was confirmed was designated as gestational day 0 (GD0).

At the end of the mating period, 33 females were randomly allocated into two experimental groups: a control group (*n* = 13) and a benzydamine-treated group (*n* = 20). In accordance with recommendations for reproductive toxicity studies requiring a minimum of 20 animals per treatment group, the benzydamine group was assigned the minimum recommended number, while the remaining animals were included in the control group. A separate pregnancy confirmation procedure was not performed, as abdominal palpation is typically feasible only after approximately gestational day 10, which would have interfered with the study protocol and treatment schedule. Therefore, treatment was administered to all females with confirmed mating. In this study, all mated females subsequently proved to be pregnant.

All experimental procedures were conducted in compliance with Directive 2010/63/EU on the protection of animals used for scientific purposes, following approval by the Scientific Research Ethics Committee of “G.E. Palade” UMPhST Targu Mures (Approval no. 2073 din 15 February 2023) and the Veterinary Health Directorate.

Females were housed individually. From post-mating day 2 (considered GD2), animals were treated by oral gavage every other day to simulate intermittent exposure. The Control group (C, *n* = 13) received distilled water in a volume equivalent to that administered to the treated group, while the Benzydamine group (B, *n* = 20) was given benzydamine at 261 mg/kg body weight. As no comparable studies are available in the literature, dose selection was based on a human exposure dose of 3000 mg (a high dose used for psychotropic purposes) and calculated using the body surface area–based interspecies dose conversion method described by Nair and Jacob [42].

To evaluate benzydamine toxicity, pregnant rats were subjected to cesarean section on gestational day 21 under inhalational isoflurane anesthesia (the final benzydamine dose was administered one day before C-section). Uterine contents were examined macroscopically, after which the dams were euthanized by anesthetic overdose. Maternal blood and placental samples were collected during the experiment.

Macroscopic examination of the uterus included assessment of the number of fetuses, fetal viability, presence of external malformations, uterine resorptions, and fetal body weight at term. All parameters were recorded individually per dam and fetus and analyzed statistically. Resorptions were classified as early (embryonic remnants only) or late (embryonic and placental remnants present), based on the presence of placental tissue in the uterine horns.

### 4.2. Maternal Oxidative Stress

Plasma was selected as the biological matrix for the evaluation of oxidative stress biomarkers in pregnant rats. MDA was measured as a stable end-product of lipid peroxidation, while XO activity was assessed as a circulating enzymatic source of reactive oxygen species.

Plasma MDA levels were quantified using a high-performance liquid chromatography (HPLC) method coupled with ultraviolet (UV) detection [43]. Xanthine oxidase activity was determined using a colorimetric assay, employing a commercially available Xanthine Oxidase Activity Assay Kit (Catalog No. MAK078, Sigma-Aldrich, St. Louis, MO, USA), according to the manufacturer’s instructions.

### 4.3. Maternal Serum Biochemical Parameters

Maternal serum biochemical parameters, including albumin (ALB), total protein (TP), globulins (GLOB), albumin/globulin ratio (A/G), total bilirubin (TB), aspartate aminotransferase (AST), alanine aminotransferase (ALT), gamma-glutamyl transferase (GGT), alkaline phosphatase (ALP), creatinine (CREA), blood urea nitrogen (BUN), glucose (GLU), total cholesterol (TC), calcium (Ca), phosphate (PHOS), potassium (K), sodium (Na), and the BUN/CREA ratio, were measured using the Element RC Clinical Chemistry Analyzer (Scil) with the General Health Rotor (scil animal care company GmbH, Viernheim, Germany).

### 4.4. Statistical Analysis

Statistical analysis was performed using GraphPad Prism 10 for macOS (version 10.6.1, GraphPad Software, San Diego, CA, USA). Data are expressed as mean ± standard deviation (SD). Comparisons between two independent groups were carried out using Student’s *t*-test. Differences were considered statistically significant at a *p*-value < 0.05.

## 5. Conclusions

The significant reduction in mean fetal weight observed in the benzydamine-exposed group suggests a potential fetotoxic effect manifested as impaired intrauterine growth. Although benzydamine exposure did not result in statistically significant changes in maternal biochemical parameters, it was associated with notable fetal and placental pathological alterations, as well as significantly increased plasma MDA levels, supporting the presence of biologically relevant developmental toxicity and enhanced lipid peroxidation under the experimental conditions. Although our findings suggest a potential role for oxidative stress in fetal growth restriction induced by benzydamine, further studies using additional oxidative stress markers are needed to strengthen this mechanistic link.

## Figures and Tables

**Table 1:** Benzydamine influence on fetal development and viability.

Group	Number of Litters	Number of Fetuses	Mean Fetal Weight ± SD (g) [Weight Range]	*p* (C vs. B)	Resorptions/ Group	Dead Fetuses/ Group
Control (C)	13	94	5.05 ± 0.45 [3.51–6.40]	*p* < 0.05	3	1
Benzydamine (B)	20	179	3.83 ± 1.60 [1.00–6.48]		19	45

Data are expressed as mean ± SD. Statistical comparisons were performed using Student’s *t*-test.

**Table 2:** Biochemical parameters following benzydamine treatment: observed trends without statistical significance.

Parameter	Control		Benzydamine		B vs. C *	Parameter	Control		Benzydamine		B vs. C *
	Mean	Median	Mean	Median			Mean	Median	Mean	Median	
ALB (g/dL)	3.68 ± 0.46	3.7	3.67 ± 0.71	3.5	↓	Crea (mg/dL)	0.35 ± 0.10	0.3	0.37 ± 0.11	0.3	-/↓
TP (g/dL)	5.48 ± 0.46	5.55	5.53 ± 0.78	5.45	↓	BUN/Crea	34.35 ± 28.09	18.03	22.81 ± 15.27	16.6	-/↓
GLOB (g/dL)	1.79 ± 0.31	1.8	1.86 ± 0.30	2	↑	Glucose (mg/dL)	137.54 ± 24.41	142.97	134.27 ± 21.34	133.305	↓
A/G	2.08 ± 0.4	1.955	2.02 ± 0.54	1.905	↓	TC (mg/dL)	82.03 ±17.73	78.34	95.22 ± 23.77	88.005	↑
AST (U/L)	82.23± 26.67	77	80.75 ± 51.26	66	↓	Ca (mg/dL)	9.55 ± 0.64	9.52	10.00 ± 1.65	9.605	↑
ALT (U/L)	70.69 14.84	73	77.55 ± 26.83	69.5	↓	PHOS (mg/dL)	4.94 ± 1.22	5.07	5.61 ± 1.32	5.28	↑
ALP (U/L)	186.08 ± 47.43	175	185.29 ± 95.65	163	↓	K (mmol/L)	5.65 ± 0.43	5.59	5.89 ± 0.93	5.64	↑
BUN (mg/dL)	18.31 ± 2.48	18.26	20.60 ± 5.27	20.075	↑	Na (mmol/L)	148.06 ± 1.12	148.3	148.85 ± 2.27	148.15	↓
BUN/ALB	5.00 ± 0.75	5.07	5.54 ± 1.04	5.72	↑						

Values are represented as mean ± SD; ↑ increased compared with control; ↓ decreased compared with control; - no consistent change. ALB = albumin, TP = total protein, GLOB = globulins, A/G = albumin/globulin ratio, AST = aspartate aminotransferase, ALT = alanine aminotransferase, ALP = alkaline phosphatase, CREA = creatinine, BUN = Blood Urea Nitrogen, TC = total cholesterol, Ca = calcium, PHOS = phosphate, K = potassium, Na = sodium; * tendencies (B vs. C) were assessed based on group medians and overall distribution patterns rather than solely on mean values, given the variability of the data.

## Data Availability

The original contributions presented in this study are included in the article material. Further inquiries can be directed to the corresponding authors.

## Abbreviations

The following abbreviations are used in this manuscript:

OTC	Over the counter
NSAID	Nonsteroidal anti-inflammatory drug
MDA	Malondialdehyde
XO	Xanthine oxidase
SGA	Small for gestational age
ROS	Reactive oxygen species
